# Non‐Invasive Estimation of Short‐Term Changes of Transpiration Using a Combination of 3D Imaging and Energy Balance Modelling

**DOI:** 10.1111/pce.70564

**Published:** 2026-04-21

**Authors:** Simon Schmitz, Andreas Fricke, Hartmut Stützel

**Affiliations:** ^1^ Institute of Horticultural Production Systems, Vegetable Systems Modelling Section Leibniz Universität Hannover Hannover Germany

Conventional approaches to measuring stomatal conductance (*g_s_*) and transpiration often rely on instruments that interfere with plant physiology. Porometers, for example, restrict natural leaf movement, apply pressure, and introduce dry airflow that can alter stomatal behaviour, thereby reducing the relevance of such measurements. Prior studies report discrepancies among devices attributable to such interferences (Toro et al. 2019). To minimise artefacts, transpiration should be estimated remotely without physical contact, which theoretically can be achieved via a thermal leaf energy‐balance approach that infers *g_s_* from leaf temperature, radiative load, and boundary‐layer terms. In this study, we combine 3D plant models, light interception models, and thermal imaging to solve the energy‐balance equation of individual leaves, estimating transpiration entirely remotely.

Approaches to estimate stomatal conductance based on the energy‐balance equation were developed recently to aid phenotyping of plantss. Most methods either imposed rapid changes in air humidity to perturb transpiration and, consequently, leaf temperature (Driever et al. 2023), or relied on ‘dry’ and ‘wet’ reference surfaces (as in Leinonen et al. 2006) to compute stress indices (Vialet‐Chabrand and Lawson 2020). These methods require reference materials to assess surface temperatures under maximum and zero transpiration, showing the effect of longwave radiation. However, reference‐material methods were constrained by heterogeneity in light interception caused by variation in leaf angle and orientation, because reference surfaces could not reorient like real leaves (Zhang et al. 2025).

In this study, we addressed this challenge by using thermal imaging and 3D photogrammetry to capture leaf temperature and geometry noninvasively, allowing parameter estimation for each leaf individually.

Leaf energy balance depends mainly on two components: input from absorbed radiation and loss through evaporative cooling (transpiration). Under steady‐state conditions, inputs and losses sum up to zero. Changes in radiation or stomatal dynamics modify energy fluxes, altering the energy stored in leaves and, consequently, leaf temperature (Vialet‐Chabrand and Lawson 2019). Using the measured light field and each leaf's area and orientation (Supporting Information S2: Equation S14), we compute absorbed radiation. Leaf temperature provides the storage term and boundary‐layer resistance is derived from leaf size and air speed (Albrecht et al. 2020). With all other terms specified, the leaf energy balance has a single remaining unknown, the stomatal resistance *r_s_* (Leinonen et al. 2006):

(1) $$r_{s}=-\rho c_{p}r_{\mathrm{HR}}\frac{s\left(T_{L}-T_{A}\right)+\delta_{e}}{\gamma \left(\left(T_{L}-T_{A}\right)\rho c_{p}-r_{\mathrm{HR}}R_{n}\right)}-r_{\mathrm{va}}.$$

Here, *ρ* is the density of air (kg m^−3^), *c*_*p*_ is the specific heat capacity of air (J kg^−1^ K^−1^) and *r*_*HR*_ is the parallel resistance to heat and radiative transfer on the leaf surface (s m^−1^), *s* is the slope of the curve relating saturating water vapour pressure to temperature (Pa °C^−1^). *T*_*L*_ and *T*_*A*_ are leaf and air temperatures (°C), respectively, *δe* is air vapour pressure deficit (Pa), *γ* is the psychrometric constant (Pa K^−1^) and *r*_*va*_ is the boundary layer resistance to water vapour (s m^−1^) (Supporting Information S2: Equation S1). The net radiative energy *R*_*n*_ in the energy‐balance term was obtained from the same 3D light interception model, which integrates measured direct and lateral scattered irradiance (W m^−2^) (Supplement Material and Methods, File S2). Stomatal conductance *g_s_* (m s^−1^) is the inverse of stomatal resistance *r_s_* (s m^−1^).

To experimentally obtain a wide range of *g_s_* values, we grew eggplant (*Solanum melongena* L.) plants in hydroponic units in growth chambers under four sets of environmental conditions (Table 1). Thirty‐day‐old plants (4–5‐leaf stage) were placed on balances (Supplementary Materials and Methods, File S2). Units were sealed with plastic film to minimise evaporation. Mass loss attributable to transpiration was logged automatically every 30 s. To induce short‐term changes in stomatal conductance, we imposed an acute osmotic stress by delivering a saline NaCl solution with high electrical conductivity (60 mS cm^−1^) to the root zone, producing a steep drop in root osmotic potential. This created rapid physiological and morphological responses that altered incident irradiance at the leaves, leaf temperature, and consequently energy balance, stomatal conductance and transpiration. We chose this stressor for operational simplicity. Any perturbation that modifies transpiration dynamics and thus gas exchange could have served our purpose.

**Table 1:** Environmental treatments as combinations of light intensity, temperature and humidity variation inside of the experimental chamber.

Environmental treatment (ET)	Abbreviation (light‐temperature‐humidity)	PAR intensity at leaf level (μmol m^−2^ s^−1^)	Air temperature (°C)	Relative humidity (%)
ET 1	**L‐T**+**H**+	170	27	60
ET 2	**L**+**T**+**H**+	580	27	60
ET 3	**L**+**T**+**H‐**	580	27	30
ET 4	**L**+**T‐H‐**	580	17	30

Each leaf was monitored with a 3D camera system and thermal camera simultaneously, obtaining leaf angles, light interception and leaf temperature, allowing us to estimate *g_s_* using Equation (1) and transpiration:

(2) $$E_{t}=A*\rho_{w}*g_{v}\left(C_{\mathrm{vs}}-C_{\mathrm{va}}\right)=\frac{A*\rho_{w}*\frac{1}{r_{s}}*g_{\mathrm{va}}}{\frac{1}{r_{s}}+g_{\mathrm{va}}}\left(C_{\mathrm{vs}}-C_{\mathrm{va}}\right).$$

The total transpiration of a leaf, *E*_*t*_ (kg s^−1^), is the product of the total conductance to water vapour from the mesophyll to the atmosphere, *g*_*v*_ (m s^−1^), calculated from the estimated stomatal resistance *r*_*s*_ (s m^−1^) and the boundary layer conductance *g*_*va*_ (m s^−1^), the difference between water vapour concentration inside the leaf *C*_*vs*_ (dimensionless), and in surrounding air *C*_*va*_ (dimensionless), the leaf area *A* (m^2^), and the density of water *ρ*_*w*_ (kg/m^3^) (Jones 1992). Estimated stomatal conductance was obtained from leaf energy balance calculation (Equation 1). Boundary‐layer conductance was computed from measured wind speed and leaf dimensions (leaf area, length, width) extracted from structure‐from‐motion 3D reconstructions (Supporting Information S1: Equation S6; Grace et al. 1980). Transpiration was then calculated for each leaf at each thermal 3D imaging time point, and whole‐plant transpiration for comparison with gravimetric logs was the sum of all per‐leaf estimates.

As a non‐invasive approach, we evaluated the plausibility or our model derived stomatal conductance (Equation 2) indirectly by comparing calculated and measured whole plant transpiration. We emphasise that this is not a direct validation of *g_s_*. Rather, the close agreement between modelled and measured transpiration across the wide range of environmental treatments, both stressed and non‐stressed, provides confidence that the inferred *g_s_* is realistic.

RGB and thermal images acquired before, during, and after stress application enabled dynamic tracking of leaf position and temperature (Supplementary Material and Methods, File S2).

As expected, osmotic stress application had immediate effects on morphology and physiology. While control leaves maintained an angle of around 110° throughout, osmotic shock induced immediate turgor loss and drooping in all environments except one (Figure 1A,B). Leaf angles recovered to pre‐stress positions within 1 h, indicating adaptation to the osmotic shock and restoration of turgor. Only environment 4 (high light, low air temperature and low humidity) maintained turgor during stress. Angle shifts were most pronounced in older leaves, which drooped and reduced light interception; younger leaves better preserved structure and turgor (Supporting Information S1: Figure S2). These angle changes also altered incident irradiance at the leaf surface (Supporting Information S1: Figure S3).

**Figure 1:**
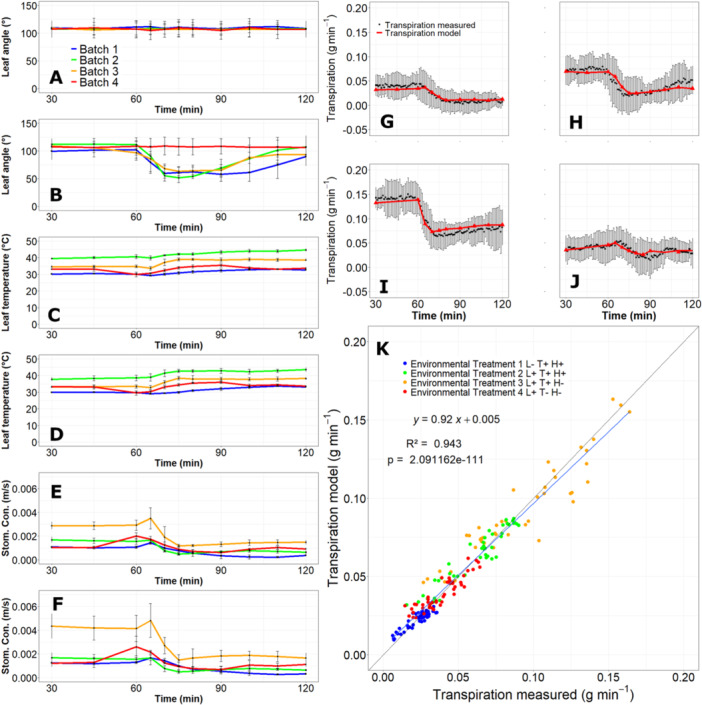
Leaf angles of eggplant under different environmental conditions under control conditions (A) and their response to rapid osmotic stress application (B), *n* = 4. Leaf temperatures of older leaves (C, leaf ranks 1 + 2) and younger leaves (D, leaf ranks 4 + 5) in response to rapid osmotic stress application, *n* = 4. Estimated stomatal conductance of older leaves (E, first and second to develop, leaf rank 1 + 2) and younger leaves (F, leaf ranks 4 + 5) in response to rapid osmotic stress, *n* = 4. Comparison of measured and model estimated transpiration for individual sample plants in four environmental conditions (G: ET 1, H: ET 2, I: ET 3 and J: ET 4). Error bars on the measured transpiration show scale standard deviation (see Supplement Material and Methods), File S2), *n* = 1. Correlation between estimated and measured transpiration across all environmental treatments, pooling individual data points of all experimental plants (as seen in G–J), *n* = 4 (K). The abbreviation code denotes factor levels as L± (light), T± (temperature) and H± (humidity), where ‘+’ indicates high and ‘−’ low (Table 1). All stress applications at 60 min with a 60 mS/cm NaCl solution.

These morphological responses coincided with increases in leaf temperature, consistent with altered water fluxes and stomatal regulation after stress. Across environments, plants showed a uniform rise in leaf temperature following osmotic stress, regardless of initial temperature (Supporting Information S1: Figure S4). This response held across leaf ages, encompassing older (Figure 1C) and younger (Figure 1D) leaves.

Stomatal conductance estimated with our method followed the same pattern, dropping rapidly after osmotic shock in both older (Figure 1E) and younger (Figure 1F) leaves (Supporting Information S1: Figure S5). We estimated no stomatal conductance recovery to pre‐stress conditions over the time course of stress exposure.

Model‐estimated and gravimetrically measured transpiration showed identical time courses across all four environmental conditions (Figure 1G–J). Transpiration rates did not recover to the same extent as leaf turgor, indicating long‐term effects of the osmotic shock. Across environments and time points, correlation between model estimated and measured whole‐plant transpiration was high (Figure 1K).

In this study, stomatal conductance (*g_s_*) is a model‐derived quantity inferred from the same physically constrained framework and model (leaf temperature, boundary‐layer conductance and vapour pressure deficit). Since we did not measure g_s_ directly, we cannot validate g_s_ directly. Instead, we used a non‐invasive check via transpiration. Model predictions closely tracked measured transpiration across the four controlled environments. This agreement increases confidence that the inferred g_s_ is realistic, while we acknowledge that transpiration agreement alone is not a rigorous validation and cannot fully rule out compensating errors.

Our study demonstrated the potential of our approach to estimate transpiration accurately by combining 3D imaging and thermography with physiological modelling without the use of reference materials that imitate real leaves. This remote approach enables simultaneous assessment of morphological and physiological responses to stress, yielding a more integrated view on plant transpiration and gas exchange. In contrast to chamber and porometer measurements or IR methods requiring wet and dry references or calibration plates, our workflow is reference‐free. Absorbed shortwave radiation is derived from measured irradiance and a 3D reconstruction of leaf geometry, with no external reference materials.

Moreover, remote measurements avoid continuous pressure from clamp‐on porometers, permitting long‐term observation and capture of rapid stress responses without sustained damage or microclimate artifacts. Further, the approach is not limited by any clamp on sensors and as such enables multi‐leaf tracking. Applied to crop canopies, this approach could improve understanding of canopy processes that influence productivity and enable remote estimation of canopy transpiration. Future research could further improve by replacing our strong saline solution stress by gradual soil drying to depict a more realistic and natural stress while testing the approach under long‐term conditions. Recent studies indicate that, with rising atmospheric CO_2_ concentrations, breeding for reduced stomatal conductance could increases WUE without affecting photosynthetic capacity (Srivastava et al. 2024). As such, remote systems for high‐throughput plant phenotyping (HTP) are required to scan vast quantities of plants. We see a potential use of our system for such purposes to quickly estimated whole plant and individual leaf transpiration, as initial image capturing is very fast. A large bottleneck in our work was 3D model generation speed and manual extraction of leaf parameters from these 3D models. Both could be streamlined with more automated software, possibly including neural network solutions.

## Funding

The authors have nothing to report.

## Conflicts of Interest

The authors declare no conflict of interest.

## Supporting information

Supporting Figure

Supporting Material

## Data Availability

The data that support the findings of this study are available from the corresponding author upon reasonable request.
