# Supplementary material for: Non‐Invasive Estimation of Short‐Term Changes of Transpiration Using a Combination of 3D Imaging and Energy Balance Modelling
Source: Plant Cell Environ. 2026 Apr 21;49(8):5470–3. doi: 10.1111/pce.70564 (PMC13353650; doi:10.1111/pce.70564)
Supplement: Supplementary file 1 — Supporting Figure [file PCE-49-5470-s002.docx]

**Supplemental Results**

**Leaf area**

Regression of 3D-derived leaf area against leafarea scanner measurements showed an excellent fit (R² ≈ 1.00) and a slope of 0.998 indicating a very high agreement between leaf scanner and 3D model leaf area measurements (Figure S1).


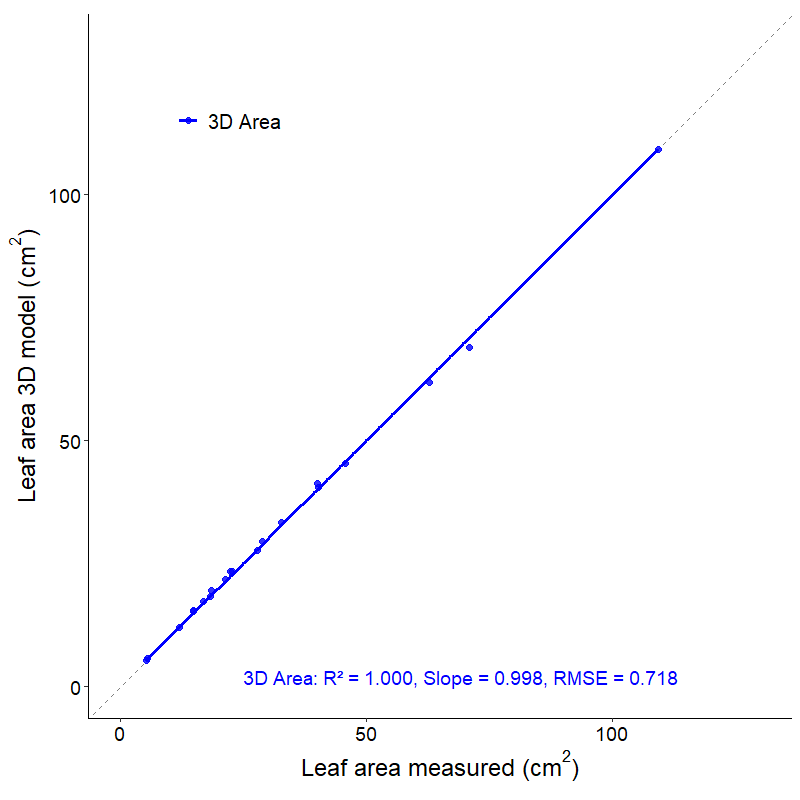


Figure S1: Comparison of measured to model calculated leaf area. 3D Area (blue) describes the 3D model leaf area linear fit, the dotted line describes the 1:1 identity, n=16.


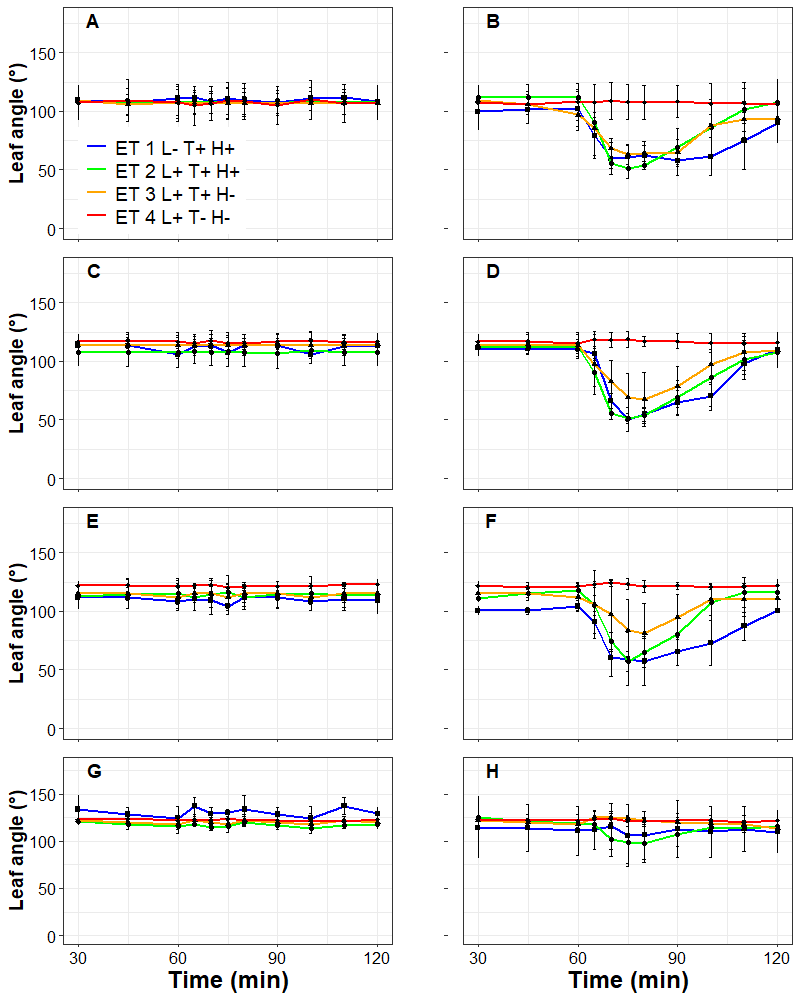


Figure S2: Leaf angles (inclination relative to gravity (0° = vertical down; 90° = horizontal) of eggplant leaves before and after the imposition of a short-term osmotic stress under different environmental treatments (ET). Stress application at 60 minutes (A: Leaf rank 1 control, B: Leaf rank 1 osmotic stress, C: Leaf rank 2 control, D: Leaf rank 2 osmotic stress, E: Leaf rank 3 control, F: Leaf rank 3 osmotic stress, G: Leaf rank 4 control, H: Leaf rank 4 osmotic stress). L, T and H indicate the environmental conditions. L+ 580 μmol m^-2^ s^-1^ PAR, L- 190 μmol m^-2^ s^-1^ PAR, T+ 27°C, T- 17°C, H+ 60% rH, H- 30% rH. Leaves are labeled by order of emergence on the plant (First leaf to emerge = “Leaf rank 1”). Error bars indicate standard deviation, n=4.


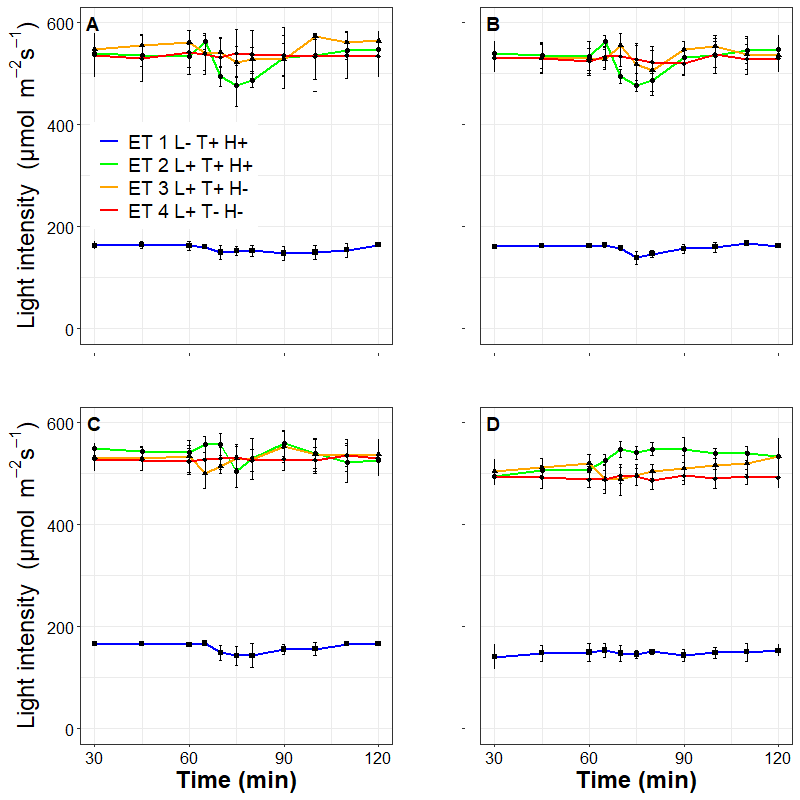


Figure S3: Incident light intensities on individual leaves before and after the imposition of a short-term osmotic stress under different environmental conditions. Stress application at 60 minutes (A: Leaf rank 1, B Leaf rank 2, C: Leaf rank 3 D: Leaf rank 4). L, T and H indicate the environmental conditions. L+ 580 μmol m^-2^ s^-1^ PAR, L- 190 μmol m^-2^ s^-1^ PAR, T+ 27°C, T- 17°C, H+ 60% rH, H- 30% rH. Leaves are labeled by order of emergence on the plant (First leaf to emerge = “Leaf rank 1”). Error bars indicate standard deviation, n=4.

**Leaf temperatures**

Under pre-stress equilibrium, leaf temperatures were stable. The response to osmotic stress was uniform across environments and leaf ages (Fig. S4). Some leaves showed a brief initial decrease, but all subsequently rose to a new quasi-steady temperature within 10–20 minutes. Absolute leaf temperatures were clearly influenced by light, air temperature, and humidity. Environmental treatment (ET) 2 (L+ T+ H+) exhibited the highest leaf temperatures, whereas ET 1 (L− T+ H+) showed the lowest, consistent with greater radiative loading under high light at comparable air temperature and humidity (L, T, H denote incident light, air temperature, and relative humidity; +/− indicate high/low levels).


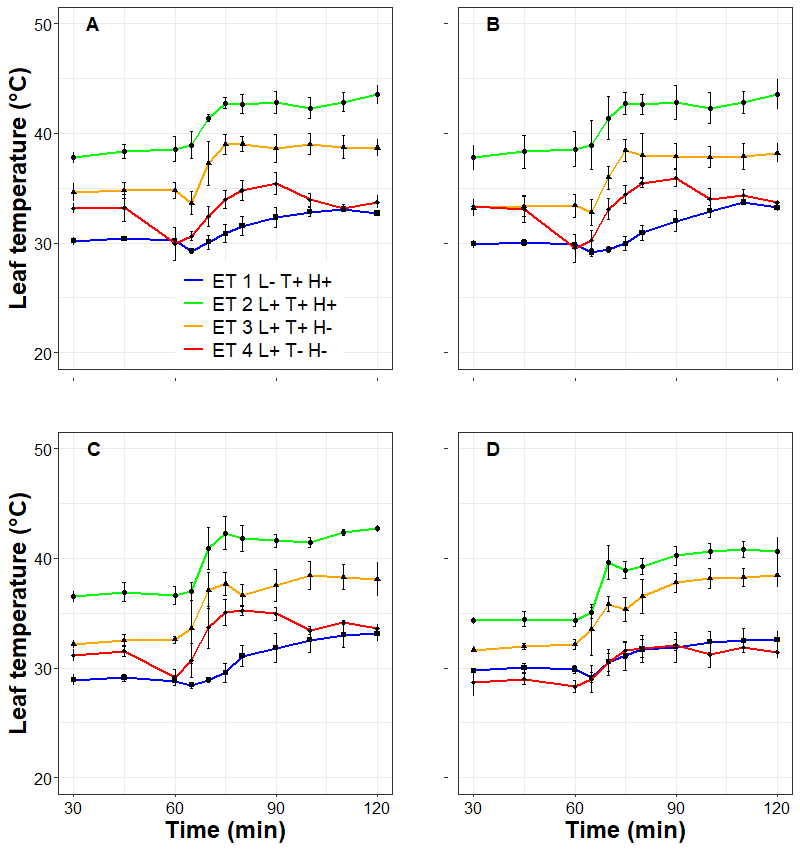
Figure S4: Average leaf temperature of leaves 1-4 before and after the imposition of a short-term osmotic stress under different environmental conditions. Stress application at 60 minutes (A Leaf rank 1, B Leaf rank 2, C Leaf rank 3 and D Leaf rank 4). L, T and H indicate the environmental conditions. L+ 580 μmol m^-2^ s^-1^PAR, L- 190 μmol m^-2^ s^-1^ PAR, T+ 27°C, T- 17°C, H+ 60% rH, H- 30% rH. Leaves are labeled by order of emergence on the plant (First leaf to emerge = “Leaf rank 1”). Error bars indicate standard deviation, n=4. Stress introduction at 60 minutes.

**Stomatal conductance**

Under pre-stress equilibrium, model estimated leaf-level stomatal conductance was approximately constant. Baseline conductance depended on both environmental conditions and leaf age, with younger leaves exhibiting higher values than older leaves. Following osmotic stress, some leaves showed a brief, sharp transient increase. Subsequently, all leaves declined uniformly over ~15–20 minutes to a new, lower quasi-steady state that was maintained until the end of the experiment (Fig. S5). All stomatal conductance values were estimated from our model.


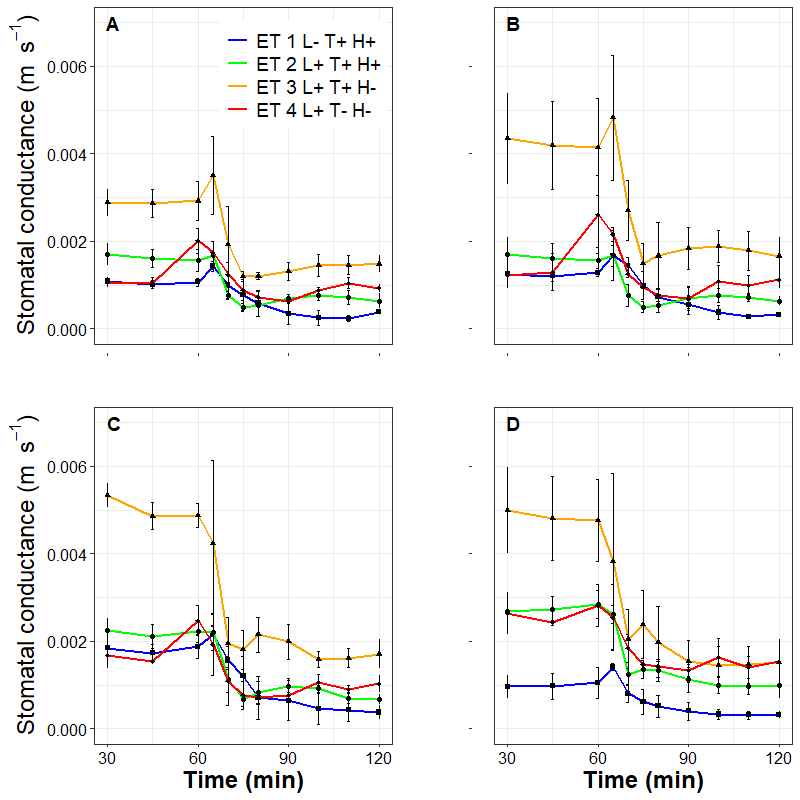


Figure S5: Estimated stomatal conductance of leaves 1-4 (A: Leaf rank 1; B: Leaf rank 2; C: Leaf rank 3; D: Leaf rank 4) before and after osmotic stress introduction which occurred at 60 minutes. L, T and H indicate the environmental conditions. L+ 580 μmol m^-2^ s^-1^ PAR, L- 190 μmol m^-2^ s^-1^ PAR, T+ 27°C, T- 17°C, H+ 60% rH, H- 30% rH. Leaves are labeled by order of emergence on the plant (First leaf to emerge = “Leaf rank 1”). Error bars indicate standard deviation, n=4. Stress introduction at 60 minutes.
