# Supplementary material for: Non‐Invasive Estimation of Short‐Term Changes of Transpiration Using a Combination of 3D Imaging and Energy Balance Modelling
Source: Plant Cell Environ. 2026 Apr 21;49(8):5470–3. doi: 10.1111/pce.70564 (PMC13353650; doi:10.1111/pce.70564)
Supplement: Supplementary file 2 — Supporting Material [file PCE-49-5470-s001.docx]

**Material and Methods**

**Plant material**

Eggplant (*Solanum melongena* L.) cv. ‘Elisa F1’ (Graines Voltz, Loire-Authion, France) was grown hydroponically in a controlled-environment growth chamber at 27°C, 60% relative humidity (rH), and a 12 h photoperiod. Mean leaf-level light intensity was ~400 μmol m^−2^ s^−1^. Plants were rooted in rockwool cubes (Grodan, Roermond, The Netherlands) and fertigated three times per week with 1 g L^−1^ Ferty 2 Mega (PLANTA Düngemittel GmbH, Regenstauf, Germany); the nutrient solution had an electrical conductivity of 1 mS cm^−1^. The only light source in the chambers were ceiling mounted halogen metal vapor lamps (Osram Powerstar HQI-BT 400W / D PRO, Munich, Germany). After the initial growth phase, plants were transferred to an experimental chamber configured according to Table 1.

The experimental chamber was identical to the growth chamber except for the target light intensity, temperature, and humidity. Plants were acclimated in the experimental chamber for one day prior to stress exposure. We employed a two‑chamber approach to standardize plant developmental state and morphology across environmental treatments. All plants were produced under identical growth conditions to harmonize leaf size, number of leaves, and structural traits (e.g., specific leaf area, thickness), as well as stomatal and hydraulic capacity established during development. One day before stress application, plants were transferred to the experimental chamber to impose the target light intensity, temperature, and humidity. This timing provided short-term adaptation to the new microclimate while avoiding treatment-induced changes in structural traits (e.g., altered specific leaf area or leaf expansion rates). As a result all plants were uniform in size and other morphological parameters (like leaf thickness), which otherwise would have been affected by growth under different environmental conditions. This design increases comparability among treatments and improves the interpretability and robustness of model–data comparisons.

**Plant selection, preconditioning, and saline stress application**

For each environmental treatment, four healthy plants, uniform in size and development stage were selected (total n = 16). Plants were removed from their hydroponic trays and installed in a closed plastic box system, which fully enclosed the rockwool block while leaving stem and foliage exposed. These boxes were large enough (roughly 1 liter of volume) to to contain the rockwool pot and were wrapped with plastic foil to prevent all water loss through evaporation, leaving only the leaf system uncovered and free to transpirate. Units were then placed on the balances and allowed to acclimate to the closed-box conditions for 60 min before salinity stress application experiment.

Salinity stress was imposed by injecting 100 mL of an NaCl solution with an electrical conductivity of 60 mS cm^-1^ into the rockwool pot using a 100 mL plastic syringe (no needle). The solution was injected from above directly beneath the stem, targeting the root network within the rockwool pot to ensure rapid dispersion and an instantaneous, uniform saline shock across plants. Control plants were treated identically but received 100 mL of the standard fertilization solution (electrical conductivity 1 mS cm^−1^).

**Experimental design**

We quantified the effects of light intensity, air temperature, and rH on transpiration using four environmental treatments (ET) (Table 1). Between adjacent treatments, a single factor differed: ET 1 vs 2 (light intensity), ET 2 vs 3 (rH), and ET 3 vs 4 (air temperature). The abbreviation code denotes factor levels as L± (light intensity), T± (temperature), and H± (humidity), where “+” indicates high and “-” low. “ET” refers to the environmental treatment during the stress exposure. For example, ET 1 comprised plants transferred from the growth chamber to the experimental chamber set to 170 μmol m^−2^ s^−1^ light intensity, 27°C air temperature, and 60% rH on the day before stress.

**3D imaging and remote estimation of leaf parameters (SfM workflow)**

We quantified leaf geometry remotely using Structure‑from‑Motion (SfM) photogrammetry based on multiview photogrammetry (Snavely et al., 2008; Peng et al., 2021). At each time point, we acquired 30–40 high‑resolution images (6000 × 4000 px, 24‑bit sRGB, JPG) with a Sony Alpha 6000 (Sony, Tokyo, Japan) from viewpoints distributed around and above the plant to capture the entire leaf system (Peng et al., 2021). Image series were collected at regular intervals before, during, and after saline stress to capture baseline and dynamic responses. All images were taken under the chamber lighting at each time point. For each picture a uniformly colored background was placed below and behind the plants.

Images were processed in Agisoft Metashape (Agisoft LLC, St. Petersburg, Russia) using a standard three‑step workflow: (1) photo alignment (SfM) to estimate camera poses and generate a sparse point cloud, (2) dense point cloud reconstruction, and (3) mesh generation over the dense cloud. The resulting leaf meshes formed the basis for geometric analyses (leaf area, linear dimensions, and leaf angles).

After reconstruction, background faces were removed by color thresholding in MeshLab (Cignoni et al., 2008) using the “Select Faces by Color” tool to select background‑colored points/faces, which were then deleted.

Leaf area, linear dimensions, and leaf angles were derived from the cleaned meshes using MeshLab and Blender (Stichting Blender Foundation, Amsterdam, Netherlands). Meshes were used to compute angles relative to gravity (horizontal) and to the light direction. Leaf areas were computed on reconstructed meshes after background removal.

**Accuracy assessment**

To evaluate accuracy, 3D‑derived leaf areas were compared against measurements from a conveyor‑belt leaf area meter (LI‑3100C, LI‑COR, Lincoln, NE, USA). The 3D method showed an excellent fit to the scanner measurements; an initial overestimation from boundary background inclusion was removed by color‑based background filtering, bringing the regression slope to ~1 (see Figure S1 and Supplementary Results).

**Measurements**

The light intensity on a horizontal plane at the level of the third and fourth leaf (second leaf layer from the bottom) and the diffuse light intensity coming from the side was measured with a PAR (photosynthetically active radiation) sensor (LI-190R, LI-COR Biosciences, Lincoln, USA) and a pyranometer sensor (LI-200R, LI-COR Biosciences, Lincoln, USA). Both measurements were done with the same light meter (LI-250A, LI-COR Biosciences, Lincoln, USA). From these measurements, we extracted the PAR to Wm^-2^s^-1^ conversion factor as 0.22.

In order to predict the incoming light intensity on a leaf surface we used a light intensity model (details see below) based on leaf orientation. For this, we measured the light intensity at leaf height in a variety of directions, ranging from completely horizontal to vertical, both for the leaf angle and the azimuth angle. Measurement positions were 0° (Fully horizontal), 20°, 45°, 60° and 90° (Fully vertical). Each leaf angle therefore corresponded to a light intensity. In our growth chamber, ceiling lamps were the only sources, accordingly the highest leaf‑level irradiance occurred for horizontally oriented leaves.

We measured air speed at leaf height with a hot‑wire anemometer (PCE‑423N, PCE Deutschland GmbH, Meschede, Germany). Measurements were averaged over several minutes at each time point to reduce noise. For each measurement the hot-wire anemometer was secured on a tripod and placed next to the leaves.

Air temperature was measured by internal sensors in the experimental chamber placed next to the plant leaves and a hot-wire anemometer (PCE‑423N, PCE Deutschland GmbH, Meschede, Germany) placed next to the plant leaves.

Leaf temperatures were recorded using an infrared camera (FLIR E60bx, FLIR Systems Inc., Wilsonville, USA) and analyzed in FLIR Tools. For accurate radiometric temperatures, we set leaf longwave emissivity to 0.98 (López et al., 2012) and used air temperature as the reflected apparent temperature. Distance was kept constant across measurements. Thermal images were acquired at the same time points as the 3D images for the entire leaf apparatus. These time points were 30 and 15 minutes before stress application, directly after stress application, 5, 10, 15, 20, 30, 40, 50 and 60 minutes after stress application.

**Gravimetric transpiration measurements**

Whole‑plant water loss was quantified gravimetrically using closed hydroponic units placed on precision balances. Each plant was rooted in a rockwool cube seated in a shallow plastic container filled with nutrient solution. To suppress non‑stomatal evaporation, the container and rockwool were enclosed with a plastic bag that was sealed around the stem. The stem and foliage remained uncovered to allow transpiration. Each unit was placed on a Sartorius BP 3100 S balance (Sartorius, Göttingen, Germany) and mass was logged at 30 second intervals via Sarto Connect (Sartorius, Göttingen, Germany).

A matched blank (control) unit, consisting of an identically prepared rockwool pot with a wooden pin to mimic a stem, was run concurrently to quantify residual evaporation from the hydroponic system. Transpiration was computed as the time derivative of the plant unit’s mass after subtracting the blank’s mass loss over the same interval. To reduce sensor noise and handling artefacts, we calculated per‑minute transpiration rates using a 10‑min moving‑average window on the corrected mass time series. Periods with obvious disturbances (repositioning, application of saline stress solution) were excluded prior to smoothing.

All measurements were done in the experimental chamber under set environmental conditions as seen in Table 1.

**Stomatal conductance model**

Most of the variables of equation 1 (Main text) can be measured or calculated directly. Only the two resistances *r_va_* and *r_HR_* require further calculation. The boundary layer resistance to water vapor, *r_va_*, can be described as the inverse of the boundary layer conductance *g_va_* calculated in equation S6. Here, *Nu* is the Nusselt number (dimensionless) and *k* is the thermal diffusity of air (m^2^ s^-1^):

$r_{va}=\frac{1}{g_{va}}=\frac{d}{Nuk}$ (S1)

The parallel resistance to heat and radiative transfer *r_HR_*, is a combined resistance made up of the resistance for radiative heat transfer *r_R_* and the resistance for heat transfer through convection *r_H_* :

$r_{HR}=\frac{r_{R}*r_{H}}{r_{R}+r_{H}}$ (S2)

The resistance for radiative heat transfer, *r_R_*, is inversely related to the third power of leaf temperature *T_L_* (°C) with *σ* being the Stefan-Boltzman constant (5,67 *10^-8^ W m^-2^ K^-4^), *ρ* the density of air (kg m^-3^) and *c_p_* is the specific heat capacity of air (J kg^-1^ K^-1^):

$r_{R}=\frac{\rho c_{p}}{4\sigma T_{L}^{3}}$ (S3)

Lastly, the resistance for heat transfer through convection *r_H_* is a function of the Nusselt number *Nu* (Equation S7):

$r_{H}=\frac{\rho c_{p}d}{Nuk}$ (S4)

**Transpiration model**

Equation 2 (main text) can be calculated with the following steps. The total conductance to water vapour *g_v_* is combined of stomatal conductance, *g_s_* (m s^-1^), and boundary layer conductance, *g_va_* (m s^-1^):

$g_{v}=\frac{g_{s}*g_{va}}{g_{s}+g_{va}}$ (S5)

The boundary layer conductance *g_va_* is the product of Nusselt number *Nu* (dimensionless), thermal diffusity of air *k* (m^2^ s^-1^) and the characteristic dimension of the leaf *d* (m). For the characteristic dimension, the width of the leaf across which the airflow is traveling times 0.72 is taken (Grace et al., 1980):

$g_{va}=\frac{Nuk}{d}$ (S6)

The Nusselt number describes the ratio of convective to conductive heat transfer at a boundary and is calculated as a variation of the Reynolds number, *Re*, the kinematic viscosity of air, *v* (m^2^ s^-1^), and *k*. We assumed a laminar airflow over all leaves due to chamber airflow being introduced from the side and at a low speed, resulting in low Reynolds numbers (~200 -500). Under faster air speeds or different leaf architecture, like drooping leaves, these conditions can change to turbulent airflow, requiring an alternate calculation of the Nusselt number (S7b). In our calculation we tested an approach to switch between these calculations based on leaf turgor status, but found no significant change in transpiration estimation.

$Nu=Re^{0.5}*\left( \frac{v}{k} \right)^{0.33}$ (S7)

$Nu=0.023*Re^{0.8}* \left( \frac{v}{k} \right)^{0.3}$ (S7b)

The Reynolds number describes the ratio between inertial to viscous forces in a fluid, in this case air, and is dependent on the wind speed *u* (m s^-1^), the characteristic dimension of the leaf *d* (m) and the kinematic viscosity of the air *v* (m^2^ s^-1^).

$R_{e}=u\frac{d}{v}$ (S8)

The concentration of vapor at the leaf surface C_vs_ is equal to the ratio of saturation vapor pressure at leaf temperature, *e_s_(T_L_)* (kPa), and actual air pressure, *P_a_* (kPa) :

$C_{vs}=\frac{e_{s}\left( T_{L} \right)}{P_{a}}$ (S9)

To compute the saturation vapor pressure at the leaf surface *e_s_(T_L_)*, we used the Tetens approximation using measured leaf temperature *T_L_* (°C). This empirical form closely follows the Clausius–Clapeyron relationship and provides an accurate fit to the saturation vapor pressure of water in the 0–50°C range. The constants used here (17.502, 240.97) correspond to the widely used Buck parameterization (Buck 1981), and the factor 0.611 kPa equals *e_s_(T_L_)* at 0°C. We apply the liquid‑water formulation because leaf temperatures in our experiments were well above 0°C.

$e_{s}\left( T_{L} \right)=0.611kPaexp\left( \frac{17.502T_{L}}{240.97+T_{L}} \right)$ (S10)

We calculated the air pressure *P_a_* based on the altitude of the leaf location *L* (m):

$P_{a}=101.3kPaexp\left( \frac{-L}{8200} \right)$ (S11)

Finally, the concentration of vapor in the air, *C_va_*, is calculated similar to *C_vs_* (equation S9), but modified with a term for relative humidity *h_r_* (%):

$C_{va}=\frac{e_{s}\left( T_{A} \right)*h_{r}}{P_{a}}$ (S12)

with

$e_{s}{(T}_{A})=0.611kPaexp\left( \frac{17.502T_{A}}{240.97+T_{A}} \right)$ (S13)

All this gives us the transpiration *E* (kg s^-1^) of an individual leaf. The total transpiration of a plant is the sum of all individual leaf transpirations. This total transpiration is then compared to gravimetrically measured weight loss in a closed hydroponic system.

Biological factors like stomatal density and stomatal ratio were not considered, as our model approach only took into account energy balance terms. While these are important factors influencing transpiration and gas exchange (Tuzet et al., 2003) they do not influence our work, as we only focused on remotely observable conditions. We similarly also excluded the effect of CO_2_ concentration on stomatal aperture, instead assuming a constant atmospheric content inside our experimental chamber.

**Light interception**

The light intensity incident on an individual leaf, *I_inc_* (W m^-2^), is the sum of the light coming directly from the light source above and the light reflected by the walls of the growth chamber:

$I_{inc}=R_{d}*\left( 1-R_{\text{\%}} \right)*A_{\text{\%}}+(R_{d}*R_{\text{\%}})$ (S14)

With *R_d_* being the light intensity on a horizontal plane at leaf height (W m^-2^), *R_%_* being the ratio between the light intensity on a horizontal compared to a vertical plane (%) and *A_%_* being the ratio between the leaf area and its calculated shadow on a horizontal plane below the plant (%).

The ratio A% was calculated as the shadow of a leaf by a light source directly above the plant.

$A_{\text{\%}}=\frac{A*cos\left( \left( \theta_{1}-90 \right)*\left( \frac{\pi}{180} \right) \right)*cos\left( \left( \theta_{2}-90 \right)*\left( \frac{\pi}{180} \right) \right)}{A}$ (S15)

Which can be reduced to

$A_{\text{\%}}=sin\left( \frac{\theta_{1}*\pi}{180} \right)*sin\left( \frac{\theta_{2}*\pi}{180} \right)$ (S16)

With A being the leaf area (m^2^) and *θ_1_* and *θ_2_* being the leaf angle and the azimuth angle relative to the ground (°), respectively. We assumed, similar to previous works (Leinonen et al., 2006), that net isothermal radiation was equal to the absorbed short wave radiation. We measured both the direct, top down, light intensity and the scattered light intensity from the side, to cover both extremes for the optimal horizontal angle leaf and the minimal vertical angle leaf. These measurements were taken in the experiment chamber at average leaf height.

The radiative term Rn in the leaf energy balance was obtained from this 3D light‑interception model, which maps each leaf surface normal to the measured angular light field at leaf height (direct top‑down and laterally scattered irradiance), thereby bracketing horizontally versus vertically oriented leaves. We assumed a spectrum-weighted leaf shortwave absorbance of 80% for eggplant, consistent with typical dicot leaf optics and a constant 10% reflectance and 10% transmittance.

$\alpha=1-p-\tau$ (S17)

Here α is the absorptance of our eggplant leaves (dimensionless, estimated as 0.8), p the reflectance (dimensionless, estimated as 0.1) and τ transmittance (dimensionless, estimated as 0.1). Absorbed radiation is then the product of intercepted radiation and absorptance.

$Q_{Rn,sw}=I_{inc}* \alpha$ (S18)

With Q_Rn,sw_ (W m^-2^) as absorbed or net shortwave radiation. Total net radiation Rn (W m^-2^) is the sum of net shortwave radiation Q_Rn,sw_ and net longwave radiation Q_Rn,lw_ (W m^-2^). Net longwave radiation is calculated from the sum of incoming and outgoing longwave radiation, measured by thermal readings of light source and leaf temperatures.

$R_{n}=Q_{Rn,sw}+Q_{Rn,lw}=Q_{Rn,sw}+Lw_{in}-Lw_{out}$ (S19)

Incoming and outgoing longwave radiation, accounting for surface emission and atmospheric absorption, is computed using the Stefan-Boltzmann law.

$Lw_{in}= \sigma* \epsilon_{glass}*T_{glass}^{4}$ (S20)

$Lw_{out}= \sigma* \epsilon_{leaf}*T_{leaf}^{4}$ (S21)

Here σ is the Stefan-Boltzmann constant (5.67*10^-8^ W m^-2^ K^-4^), ε_air_ and ε_leaf_ are emissivity values for air and leaf, respectively (unitless). ε_leaf_ was estimated as 0.98. T_glass_ and T_leaf_ (K) were recorded using an infrared camera (See Measurement section), with T_glass_ measured in the experimental chambers overhead lamp compartment, which had been separated from the chamber by a glass plate. As silica glass is largely opaque to longwave radiation (Cui et al., 2013) the only source of longwave radiation is thermal emissivity of the glass itself, calculated by the Stefan Boltzmann law. We assumed a glass emissivity ε_glass_ of 0.85.

**Inaccuracy of assumptions**

Due to the nature of our research, its focus on remote estimation and easy measurement system, we make many assumptions about conditions inside our experimental chamber. Such assumptions include airflow speed and laminar conditions across all leaves, boundary layer behavior, a simplification of shortwave and longwave radiative load and measured temperature with thermographic systems. The accurate validation of these assumptions was outside our research approach. Future works could expand on these findings with more accurately designed longwave radiation measurements, emissivity measurements of leaves and experimental chamber materials, boundary layer calculations based on laminar or turbulent airflow and airflow simulations based on varying air speed in general. A direct comparison of remotely estimated stomatal conductance values to snapshot-style measurements by porometer could further validate our approach.

**References**

**Britton, C. M., & Dodd, J. D.** (1976). Relationships of photosynthetically active radiation and shortwave irradiance. *Agricultural Meteorology*, *17*(1), 1-7. https://doi.org/10.1016/0002-1571(76)90080-7

**Buck, A. L**. (1981). New equations for computing vapor pressure and enhancement factor. *Journal of Applied Meteorology and Climatology*, *20*(12), 1527-1532. https://doi.org/10.1175/1520-0450(1981)020%3C1527:NEFCVP%3E2.0.CO;2

**Cignoni, P., Callieri, M., Corsini, M., Dellepiane, M., Ganovelli, F., & Ranzuglia, G.** (2008, July). Meshlab: an open-source mesh processing tool. In *Eurographics Italian chapter conference* (Vol. 2008, pp. 129-136).

**Cui, S., Chahal, R., Boussard-Plédel, C., Nazabal, V., Doualan, J. L., Troles, J., ... & Bureau, B.** (2013). From selenium-to tellurium-based glass optical fibers for infrared spectroscopies. *Molecules*, *18*(5), 5373-5388. https://doi.org/10.3390/molecules18055373

**Leinonen, I., Grant, O. M., Tagliavia, C. P. P., Chaves, M. M., & Jones, H. G.** (2006). Estimating stomatal conductance with thermal imagery. *Plant, Cell & Environment*, *29*(8), 1508-1518. https://doi.org/10.1111/j.1365-3040.2006.01528.x

**López, A., Molina-Aiz, F. D., Valera, D. L., & Peña, A.** (2012). Determining the emissivity of the leaves of nine horticultural crops by means of infrared thermography. *Scientia Horticulturae*, *137*, 49-58. https://doi.org/10.1016/j.scienta.2012.01.022

**Peng, Y., Yang, M., Zhao, G., & Cao, G.** (2021). Binocular-vision-based structure from motion for 3-D reconstruction of plants. *IEEE Geoscience and Remote Sensing Letters*, *19*, 1-5. https://doi.org/10.1109/LGRS.2021.3105106

**Snavely, N., Seitz, S. M., & Szeliski, R.** (2008). Modeling the world from internet photo collections. *International journal of computer vision*, *80*(2), 189-210. https://doi.org/10.1007/s11263-007-0107-3

**Tuzet, A., Perrier, A., & Leuning, R.** (2003). A coupled model of stomatal conductance, photosynthesis and transpiration. *Plant, Cell & Environment*, *26*(7), 1097-1116. https://doi.org/10.1046/j.1365-3040.2003.01035.x
